# Post-Drive Standing Balance of Vehicle Passengers Using Wearable Sensors: The Effect of On-Road Driving and Task Performance

**DOI:** 10.3390/s21154997

**Published:** 2021-07-23

**Authors:** Victor C. Le, Monica L. H. Jones, Kathleen H. Sienko

**Affiliations:** 1 Department of Mechanical Engineering, University of Michigan, 2350 Hayward St., Ann Arbor, MI 48109, USA; victle@umich.edu; 2 University of Michigan Transportation Research Institute, University of Michigan, 2901 Baxter Rd., Ann Arbor, MI 48109, USA; mhaumann@umich.edu

**Keywords:** IMU, wearables, standing balance, postural stability, vehicle motion, task performance

## Abstract

Postural sway has been demonstrated to increase following exposure to different types of motion. However, limited prior studies have investigated the relationship between exposure to normative on-road driving conditions and standing balance following the exposure. The purpose of this on-road study was to quantify the effect of vehicle motion and task performance on passengers’ post-drive standing balance performance. In this study, trunk-based kinematic data were captured while participants performed a series of balance exercises before and after an on-road driving session in real-time traffic. Postural sway for all balance exercises increased following the driving session. Performing a series of ecologically relevant visual-based tasks led to increases in most post-drive balance metrics such as sway position and velocity. However, the post-drive changes following the driving session with a task were not significantly different compared to changes observed following the driving session without a task. The post-drive standing balance performance changes observed in this study may increase vulnerable users’ risk of falling. Wearable sensors offer an opportunity to monitor postural sway following in-vehicle exposures.

## 1. Introduction

Urban transportation is anticipated to transform through the development of autonomous vehicles (AVs) and other mobility solutions (e.g., ride-sharing services). These transportation alternatives have the potential to reduce traffic congestion, increase user productivity, and provide greater access to transportation to a broader population [1]. Since AV users will be passengers, the widespread adoption of mobility solutions will likely result in an increased number of on-road vehicle passengers compared to drivers. Moreover, accessibility to AVs for broader populations will increase the diversity of passengers on the road. Accessibility is especially beneficial to older adults for increasing mobility, independence, and autonomy [2]. Across all population segments, users of mobility solutions will be freed from having to drive and will be able to engage in non-driving related tasks. However, studies of simulated driving and of in-vehicle passengers on a closed test track have demonstrated that postural control can be negatively affected by motion exposure [3,4,5,6,7,8]. Control of postural sway (especially of the trunk) is crucial for maintaining upright standing balance [9]. A substantial increase in postural sway may increase the risk of falling after an in-vehicle exposure associated with mobility solutions or AVs [10,11]. Given a larger and more diverse passenger population, those already susceptible to falling (e.g., older adults) or those not accustomed to frequent transportation may encounter an increased risk of injury. Older adults with a history of previous falls are also more likely to experience subsequent falls and injuries [12]. In the worst case, the resulting injuries from a non-fatal fall can significantly impact quality of life, reduce social and physical activity, and raise medical costs [13,14,15,16]. Therefore, it is necessary to understand how passenger vehicle transport affects the control of standing balance as increased fall risk may be a significant deterrent for certain users.

In-vehicle measurements of posture and balance have been collected using various sensors such as on-board depth cameras for head and upper body posture/orientation [17,18], as well as magnetic tracking systems for the trunk position [7,19]. When measuring post-drive postural sway, laboratory-based studies of simulated driving have leveraged typical laboratory-based instrumentation (e.g., force plates [6] and passive or active motion tracking). Options for instrumentation are constrained during in-the-field or naturalistic studies as equipment must be portable to facilitate measurements immediately following the exit from the vehicle. Although cameras mounted within the vehicle allow for accurate tracking and analysis of occupants’ movements [20], they are restricted to in-vehicle data collections. Alternatively, wearable sensors are suitable for wireless data collection and enable the measurement of in-vehicle and post-drive postural sway.

Prior studies that have quantified postural sway before, during, and after exposure to driving have mainly used surrogates of on-road driving (i.e., driving simulations, head-mounted displays, fixed-base, and a 6 degree-of-freedom (DOF) motion platforms). These surrogates can be limited by technical and physical factors such as latency, graphical fidelity, and motion scaling factors [21,22,23]. Several studies have measured postural sway at the trunk or head during a simulated (driving, flight), physical driving, or gaming exposures, and some of these studies have reported significant differences relative to pre-exposure postural sway [7,19,24,25,26,27,28]. Among the existing studies that have quantified post-drive standing balance performance, only simulated driving routes have been used with participants as the drivers. Using a 6 DOF driving simulator, Keshavarz et al. (2018) [6] observed increases in the path length of drivers’ sway following a simulated drive with varying sensory cues. In another study, Reed-Jones et al. (2008) [29] investigated driver behavior using a fixed-base driving simulator and found the inverse relationship, i.e., drivers’ path velocity decreased following a simulated drive [29]. Other researchers observed increases in drivers’ post-drive sway velocity following exposure to a fixed-base driving simulator, though the differences were not statistically significant [3]. Using a head-mounted display for a simulated drive, Mourant and Thattacherry (2004) [4] observed that drivers’ time in a single-leg stance decreased following the exposure to the simulation. Overall, different metrics of standing balance have been shown to be affected by a simulated drive despite differences in experimental modalities across the studies.

To our knowledge, our previous study conducted in a passenger vehicle on a closed test track [8] is the only study that has quantified the effects of vehicle motion and task performance on passengers’ post-drive standing balance performance. The scripted route consisted of many instances of longitudinal and lateral acceleration profiles similar to those observed in naturalistic driving datasets [30,31]. We analyzed the participants’ performance on two balance exercises performed prior to and following the 20 min continuous drive in a passenger vehicle. The participants completed two driving sessions in randomized order as front-seat passengers. During one of the driving sessions, they completed a non-driving-related task that was administered on a handheld tablet. Throughout the other drive, the participants did not complete a task and rode in a standardized posture (unrestricted gaze and head orientation, hands on lap, or feet resting on heels). Following both driving sessions, passengers’ trunk postural sway increased significantly, especially when participants performed a task throughout the driving session. We observed large increases in sway velocity and path length that were consistent with some findings of previous studies in simulated driving environments [3,6].

Comparison studies between surrogates of driving environments and naturalistic on-road driving have typically focused on the fidelity of the experimental context or the validity of the occupant’s behavior. Simulator fidelity is critical as increased fidelity has been demonstrated to affect driver performance [32,33]. As it pertains to this on-road study, a lack of contextual vehicle features (e.g., vehicle seat, interior configuration, field of view, and accurate representation of vehicle motion) may influence postural sway. Physical fidelity (how a surrogate looks) and functional fidelity (how a surrogate operates) varies across different driving surrogates [34]. For example, virtual desktop vehicle simulations have reasonable functional fidelity (e.g., steering controls) but low physical fidelity (e.g., lack of vehicle cabin or accurate sensory stimuli). In-vehicle simulations use a variety of approaches to improve fidelity including motion cueing strategies [35,36], virtual environment tools [37], use of more realistic sensory cues and stimuli [38], and enhanced mechanical capability of the motion platform to generate tilts and displacements more representative of acceleration profiles experienced during naturalistic driving conditions [23]. In our previous closed test track study, passenger behavior was quantified during an in-vehicle exposure conducted on a closed test track with high physical fidelity. This in-vehicle exposure provided moderate functional fidelity given that the frequency of the vehicle events during the scripted route greatly exceeded the number of vehicle events that typically occur during naturalistic driving conditions. Additionally, a closed test track environment does not fully replicate the sensory, environmental and contextual cues, and psychological factors associated with an on-road environment that can affect occupant behavior [39]. For instance, passengers experience naturalistic driving dynamics within the context of other on-road actors and vehicles interacting in real-time traffic.

Given the multi-faceted characteristics of on-road driving, the lack of prior work, and limitations of driving simulators, it is necessary to understand how different types of vehicle motion and task performance in an on-road environment affect post-drive standing balance performance among passengers. Therefore, the objective of this on-road study was to evaluate passenger behavior directly in the actual environment of study (an in-vehicle exposure conducted on-road under realistic driving conditions) and to compare these results to those previously gathered within the surrogate environment (i.e., the closed test track). This work contributed to our understanding of the potential risks associated with passengers’ standing balance and will inform the design and implementation of future mobility solutions and testing platforms.

## 2. Materials and Methods

### 2.1. Experimental Design

In this on-road study, participants rode in the front passenger seat of a midsize sedan that was operated by a trained driver. The driving routes consisted of various driving events or maneuvers (e.g., turning, braking) under real-time driving exposure set in midday traffic throughout Ann Arbor, MI, USA. Participants were assigned to one of two routes: an urban route that consisted of neighborhood streets and main city roads (Urban), or a highway route that included lengthy passages on local freeways (Highway). The urban route consisted of the same range of vehicle speed, number, and type of vehicle maneuvers (e.g., left and right turns, braking, lane changes, and roundabouts) as the scripted route conducted on the closed test track [8]. However, the duration of exposure differed between the closed test track and on-road studies. For the closed test track study, the scripted route was 20 min in duration; in contrast, the time required to complete the same maneuvers on-road was approximately 2.5 times longer, approximately ~55 min in duration. The Highway route was designed to evaluate the effect of longitudinal acceleration control and higher vehicle speed (~65–70 mph) under conditions of minimal lateral acceleration. Supplement Materials illustrates a map of the Urban (Figure S1) and Highway (Figure S2) routes.

Two levels of task performance were used as repeated tests during the on-road routes [31]. During the *Task* condition, participants completed a series of ecologically relevant, visual-based tasks on a handheld tablet-based device throughout the duration of the driving session. Otherwise, participants were instructed to exhibit normative passenger behavior with an unrestricted gaze (*No-Task* condition). A mixed between/within participant design was used. Participants were assigned to one of the on-road routes and were tested on the route twice, with and without the task. The order of these repeated tests on the *Task* condition was randomized. In total, there were four test conditions: Urban, *Task* (UT); Urban, *No-Task* (UN); Highway, *Task* (HT); and Highway, *No-Task* (HN).

### 2.2. Participants

The participants included 106 adults (47 males and 59 females) between the ages of 18 and 89 years (34.2 ± 18.5 years). The participant sample was further stratified by age: 82 were aged < 60 years (24.5 ± 4.3 years) and 24 were aged ≥ 60 years (67.0 ± 6.9 years). Adults under the age of 60 were classified as younger adults, while those greater than or equal to 60 years old were classified as older adults. Prior to the experiment, participants were screened and self-reported that they did not have diagnosed balance disorders, heart conditions, neurological conditions, migraines, cerebral or vascular disease, and did not use medications that might affect balance or cause dizziness (e.g., antidepressants or barbiturates) that would alter their motion sickness response or post-drive balance ability. The analysis presented in this paper was part of a larger study that explored motion sickness and on-road driving. Although descriptive data on participants’ motion sickness were gathered, the effect of motion sickness on post-drive balance performance was not included in this paper.

To facilitate comparisons between the Urban and Highway routes, a non-parametric Wilcoxon rank-sum test and a chi-squared test was performed, indicating no significant differences between the two participant samples in terms of age (*p* = 0.44, *Z* = 0.77) or sex (χ^2^ = 0.1361, *p* = 0.71). Participants provided written informed consent and the study was conducted in accordance with the Declaration of Helsinki. The study was reviewed and approved by the University of Michigan Institutional Review Board (HUM00128751).

### 2.3. In-Vehicle Test Protocol

During the in-vehicle exposure, participants were asked to maintain a standardized, neutral posture in the passenger seat; more details can be found in Jones et al. [31]. Each driving session lasted until either the route was completed (55 min on average (SD = ± 4 min)) or until the participants opted to discontinue the driving session. In total, eight participants requested to stop the vehicle prior to the end of the route; however, they ended the driving session relatively close to the end of the scripted route and were able to perform the balance protocol. Participants completed a total of two driving sessions, one for each *Task* condition. The *Task* and *No-Task* driving sessions were scheduled on two separate days with a minimum of 24 h between sessions. For the *Task* condition, participants were additionally asked to complete a visual-based task administered on a handheld tablet-based device held in their lap during the drive. Participants were instructed to complete as much of the task as possible throughout the driving session and were allowed to take breaks at their own volition.

### 2.4. Balance Exercises

Participants performed a series of balance exercises immediately prior to and following the driving session in outdoor conditions beside the stopped vehicle. Two trials of each of the following three exercises that increased in difficulty were performed:

- Exercise 1: feet together/eyes open/firm support;
- Exercise 2: feet together/eyes closed/firm support; and
- Exercise 3: feet together/eyes closed/foam support, using a compliant support surface (Airex, New York, NY, USA).

**Figure 1:**
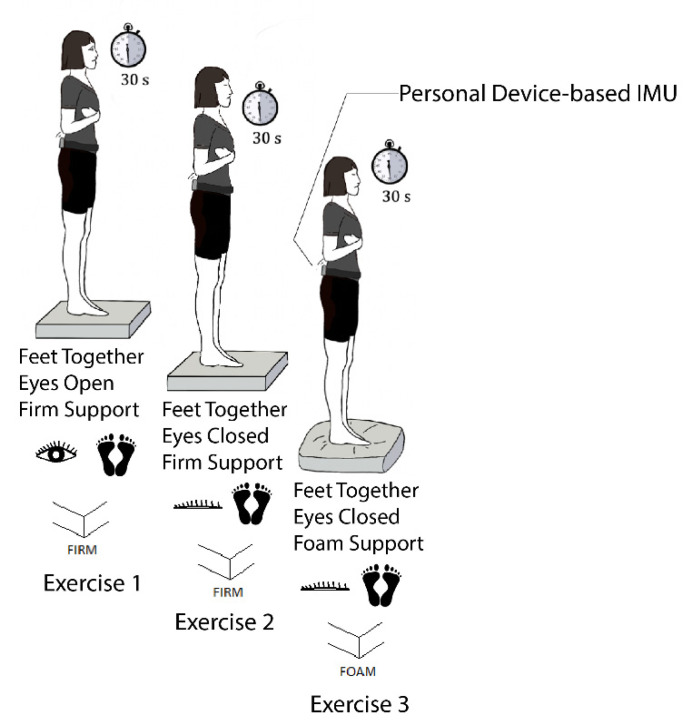
Balance exercises are shown in the order in which they were performed.

We chose these exercises because they were representative of real-world stances and visual and somatosensory scenarios (e.g., standing outside of a vehicle on paved or grassy surfaces during day and night conditions). Participants practiced this series of exercises in a laboratory setting prior to performing the pre-drive trials. During balance testing, participants were instructed to cross their arms and stand tall but avoid being stiff or tense. A visual reference target was placed at eye-level in front of the participants to control for changes in the surrounding visual field (e.g., if the participants opted to terminate the driving session before completing the route, they were asked to perform the balance exercises beside the parked vehicle). Each trial was 30 s long unless the participants either stepped out of the prescribed position or lost their balance (i.e., grabbed a nearby walker, failed to complete the exercise as described, or required intervention by a spotter to prevent a potential fall). Figure 1 illustrates this series of balance exercises.

### 2.5. Balance Measurements and Instrumentation

A surrogate smartphone (6th generation iPod Touch, 2015) secured at the participants’ lower back with an elastic waistband was used to measure anteroposterior (A/P) and mediolateral (M/L) postural sway [40,41]. Custom software installed on the smartphone extracted raw inertial measurement unit (IMU) data at a sample rate of 50 Hz from the embedded accelerometers and gyroscopes [41]. These data served as inputs into an extended Kalman filter from which tilt angle and tilt velocity were estimated. Tilt data were then processed in MATLAB (version 2020a, The MathWorks, Natick, MA, USA) and the following six balance metrics were computed [40,42]:

1. Root mean square (RMS) of trunk tilt in the A/P direction (A/P RMS);
  $$AP\;RMS=\frac{1}{N}\overset{N}{\underset{i=1}{\sum }}{\left[x_{AP}\right]}_{i}^{2}$$
2. RMS of trunk tilt in M/L direction (M/L RMS);
  $$ML\;RMS=\frac{1}{N}\overset{N}{\underset{i=1}{\sum }}{\left[x_{ML}\right]}_{i}^{2}$$
3. RMS of trunk sway velocity in the A/P direction (A/P RMS Velocity);
  $$AP\;RMS\;Velocity=\frac{1}{N}\overset{N}{\underset{i=1}{\sum }}{\left[v_{AP}\right]}_{i}^{2}$$
4. RMS of trunk sway velocity in the M/L direction (M/L RMS Velocity);
  $$ML\;RMS\;Velocity=\frac{1}{N}\overset{N}{\underset{i=1}{\sum }}{\left[v_{ML}\right]}_{i}^{2}$$
5. Path length of the trunk sway trajectory (Path Length); and
  $$Path\;Length=\overset{N-1}{\underset{i=1}{\sum }}\sqrt{{\left({\left[x_{AP}\right]}_{i+1}-{\left[x_{AP}\right]}_{i}\right)}^{2}+{\left([x_{ML}\right]}_{i+1}-{\left[x_{ML}\right]}_{i}{)}^{2}}\;$$
6. Elliptical area which is the elliptical fit of the sway trajectory (Elliptical Area),
  $$Elliptical\;Area=\pi \mathrm{ab}=2\pi F_{0.05\left[2,n-2\right]}\sqrt{\left(s_{AP}^{2}s_{ML}^{2}-s_{AP,ML}^{2}\right)}$$
  where *N* is the number of samples; *x_AP_* and *v_AP_* are the trunk position and velocity in the A/P direction, respectively; *x_ML_* and *v_ML_* are the trunk position and velocity in the M/L direction, respectively; *s_AP_* and *s_ML_* represent the standard deviation of the A/P and M/L trunk positions, respectively; *F*_0.05[2,n−2]_ is the *F* statistic at 95% confidence for a bivariate distribution; and *s_AP,ML_* is the covariance of the A/P and M/L trunk positions.

As expressed by the equations above, RMS was calculated by taking the square root of the average of the squared tilt values. To compute the Elliptical Area of sway, a 95% confidence ellipse was fit around the tilt values for each trial before computing the area [41,42]. Path Length was computed by summing the Euclidean distance between consecutive samples of the A/P and M/L tilt angles [42]. We also computed the RMS of the acceleration signals for Exercise 1 to directly compare with prior work that measured trunk sway as a function of age and other pathologies [43,44,45,46]. The methods for computing the RMS of the A/P acceleration signal were consistent with those used by Moe-Nilssen and Helbostad (2002) [9], Kosse et al. (2015) [47], and Mancini et al. (2012) [48]. These methods are further detailed in Le (2021) [49].

### 2.6. Data Analysis

In order to make comparisons between different groupings of the data, we only considered data from balance trials that were completed for the full 30 s. Out of 2544 pre- and post-drive trials, 29 trials were excluded from the subsequent analysis due to either step-outs or participants’ inability to safely complete the exercise. Twenty-four trials were excluded due to environmental factors during the balance exercises (e.g., windy conditions). Lastly, 188 trials were excluded due to missing data, resulting in a total of 2303 trials included in the analysis. Given that the data were non-normal, non-parametric statistical tests were used for all statistical comparisons.

#### 2.6.1. On-Road Analysis

Due to individual intra-variability in pre-drive balance performance, we normalized the post-drive measurements to analyze relative changes in the balance metrics across participants for each balance exercise [50,51]. Analysis of the normalized changes in balance metrics allowed for more direct comparisons between the on-road route and *Task* conditions. To compute a normalized change, participants’ post-drive measurements were divided by the average of their pre-drive measurements for each balance exercise. To isolate the effect of the driving session, the first post-drive measurement and the average of the pre-drive measurements were used.

Firstly, to assess the effect of drive on standing balance performance, Wilcoxon signed-rank tests were performed to determine if post-drive measurements increased relative to participants’ pre-drive measurements for Exercises 1–3, while collapsed across covariate descriptors. Normalized balance metrics were analyzed to determine if they were significantly different from a value of 1 that represented no change from pre- to post-drive. Additionally, statistical analysis of these normalized balance metrics was performed between the on-road route and *Task* conditions for each exercise. Specifically, Wilcoxon signed-rank and rank-sum tests compared the pre-drive and post-drive balance metrics for Exercises 1–3 within and across the Highway, *Task* (HT); Highway, *No-Task* (HN); Urban, *Task* (UT); and Urban, *No-Task* (UN) conditions. Due to the number of balance exercises, a Bonferroni correction factor or adjusted alpha level of 0.0167 per test (0.05/3) was applied for these comparisons.

We also examined the changes in post-drive standing balance performance across the two post-drive trials. In particular, we focused on Exercise 3 because it was the most challenging balance exercise and exhibited the largest changes in post-drive standing balance performance. Similar to the analysis in previous sections, balance metrics were grouped by the *Task* condition. In order to compare across participants, the balance metrics were normalized by dividing the second post-drive balance measurement by the first post-drive balance measurement. This normalization captured the changes in balance performance relative to the first trial of Exercise 3 following a driving session. Wilcoxon signed-rank tests were used to determine if the relative changes were significantly different from a value of 1 that represented no change from the first trial to the second trial. Wilcoxon rank-sum tests were also used to investigate differences across the *Task* conditions. These statistical comparisons were evaluated at a level of significance of 0.05.

#### 2.6.2. Comparative Analyses

Given that the Urban route was a scaled version of the scripted route used during the closed test track study, we compared the post-drive standing balance metrics across these conditions [8,31]. Although full details can be found in Jones et al. (2019) [31] and Le et al. (2020) [8], a brief description is provided here. Fifty adults participated in a similarly designed experiment. The previous closed test track study consisted of a concentrated 20 min driving exposure on a controlled scripted route [8,31] at two levels of acceleration (moderate or low) and repeated under the same *Task* condition. We selected one out of the two balance exercises from the closed test track study to use in this comparative analysis because it allowed for direct comparison with Exercise 3 (feet together/eyes closed/foam support) from this on-road study. To ensure valid comparisons across study populations, a non-parametric Wilcoxon rank-sum test and chi-squared test were performed across the test conditions for the closed test track study and this on-road study, finding that age (*p* > 0.1, *Z <* 1.65) and sex (χ^2^ = 9.25, *p* = 0.97) were not significantly different between study participant samples. Wilcoxon rank-sum tests (α = 0.05) were used to compare normalized changes in postural sway for Exercise 3 across conditions for the Urban and closed test track routes (Moderate and Low acceleration).

Prior to comparing the RMS of trunk acceleration to other reported values in the literature, we first established that our study participants exhibited similar baseline standing balance values with respect to the values reported by Park et al. (2016) [43]. Although their participants performed a variant of Exercise 1 (i.e., feet apart vs. feet together with eyes open on a firm support surface), prior studies have demonstrated there to be minimal differences in A/P sway between these two stances [52,53]. In comparison to the findings reported in Park et al. (2016) [43], the pre-drive values of the younger and older adult participants for our on-road study fell within the ranges specified by their respective age categories; further details can be found in Le (2021) [49].

## 3. Results

### 3.1. On-Road Driving Analysis

#### 3.1.1. Learning Effect

Prior to analysis, we observed a learning effect among pre-drive trials using a mixed model approach that informed which trials to include in our data analysis. To enable comparison with the work by Diamantopoulos et al. (2003) [54], we chose Path Length as the representative balance metric to conduct the learning effect analysis. The fixed effects were the trial number and the day of the session; participant identifiers were implemented as random variables. Estimates of the model coefficients revealed a significant difference between the first and second trials for Exercise 3 (*p* < 0.001). In contrast for Exercise 1 and Exercise 2, Path Length did not differ between trials (*p* = 0.72, 0.96, respectively). Moreover, an analysis of the practice trials performed in the laboratory revealed that the Path Length for the practice trials was significantly greater than the pre-drive trials for Exercise 3, indicating a learning effect. Therefore, for Exercise 3 only, we subsequently compared changes between the last pre-drive trial and the first post-drive trial. The analyses for Exercises 1 and 2 included all pre-drive trials.

#### 3.1.2. Effects of Route, Task Conditions, and Participant Covariates

The effects of the on-road routes, *Task* conditions, and participant covariates were also quantified using mixed models that were fit to the normalized Path Length of the sway trajectory. In addition to the fixed and random effects described in Section 3.1.1, we included additional fixed effects as categorical variables representing participant covariates (i.e., age or sex); route (Urban or Highway); and *Task* condition (*Task* or *No-Task*). For each exercise, the main effect of the route was insignificant (*p* = 0.41, *p* = 0.40, *p* = 0.34 for Exercises 1–3, respectively). Additionally, the effect of age or sex was not found to be significant. Therefore, all subsequent analyses were conducted on the combined dataset, combined across the Urban and Highway routes and the participant covariates.

#### 3.1.3. Pre-Post Drive Analysis

Table 1 outlines the results from the pre-post analysis of the normalized changes in balance metrics as a function of the *Task* condition for each exercise. Across all the exercises, there were significant increases in nearly all of the balance metrics for the *Task* condition. For the final, most difficult balance exercise (Exercise 3), we found significant post-drive increases across all balance metrics during both *No-Task* and *Task* conditions. With the exception of A/P RMS for the *No-Task* condition (*p* < 0.01, *Z =* 3.16), the *p*-values for the comparisons among the other metrics were <0.001, with the largest *Z*-statistic reported for Path Length (*Z* = 7.35) and M/L RMS sway velocity (*Z* = 7.01).

Many of the balance metrics associated with Exercise 1 were also significant. For the *No-Task* condition, M/L RMS (*p* < 0.01, *Z* = 2.62), M/L RMS sway velocity (*p* < 0.01, *Z* = 3.14), and Elliptical Area (*p* < 0.01, *Z* = 3.29) increased significantly following the driving session. Similarly, for the *Task* condition, M/L RMS sway (*p* < 0.001, *Z* = 4.04), M/L RMS sway velocity (*p* < 0.001, *Z* = 3.97), and Elliptical Area (*p* < 0.001, *Z* = 4.25) increased significantly. In contrast, for Exercise 2, fewer balance metrics increased following a driving session, with only Elliptical Area (*p* < 0.01, *Z* = 3.21) increasing significantly following a driving session for the *No-Task* condition.

#### 3.1.4. Effect of Task Conditions

For Exercises 1 and 2, the normalized balance metrics were not statistically different between the two *Task* conditions. For Exercise 3, normalized RMS sway in the M/L directions increased significantly for the *Task* condition compared to the *No-Task* condition (*p =* 0.0014, *Z* = 3.19). The median, quartiles, Z-statistics, and *p*-values associated with the statistical comparisons are presented in Table 2.

#### 3.1.5. Changes in Post-Drive Standing Balance Between Trials

To investigate the change in standing balance performance across the two post-drive trials, we analyzed the normalized balance metrics for Exercise 3 because it was the most challenging balance exercise and demonstrated the largest post-drive changes. All normalized balance metrics exhibited significant differences between the first and second trial for the *Task* condition. For the *No-Task* condition, sway velocity in the A/P (*p* < 0.001, *Z* = −5.46) and M/L (*p* < 0.001, *Z* = −5.97) directions, and Path Length (*p* < 0.001, *Z* = −7.03) decreased significantly as a function of post-drive trial number. Normalized post-drive measures of M/L RMS sway were significantly different (*p* < 0.001, *Z* = 3.35) when comparing between the *No-Task* and *Task* conditions. All statistical comparisons are shown in Table 3.

### 3.2. Comparative Analyses

**Figure 2:**
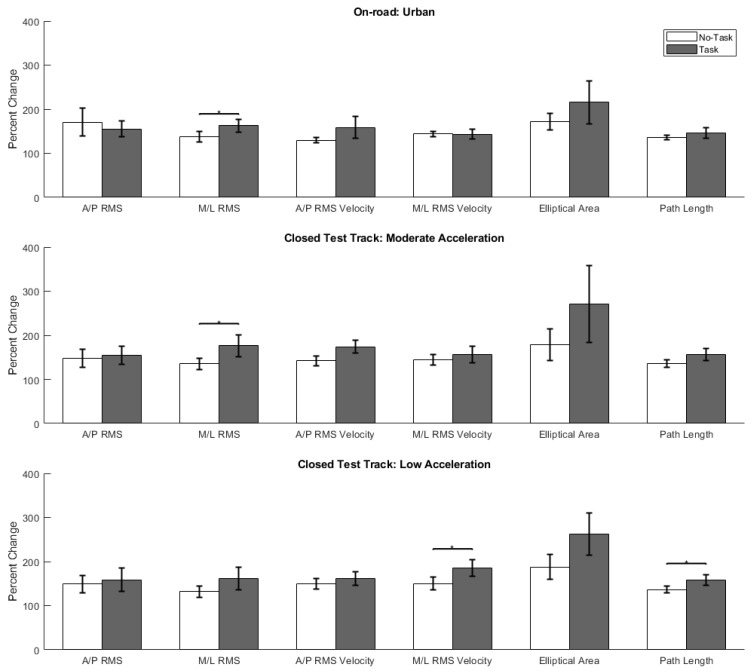
Bar plot illustrating the means and standard errors for the six normalized balance metrics for Exercise 3 (feet together/eyes closed/foam support) across studies. Abbreviations: RMS = root mean square; A/P = anteroposterior; M/L = mediolateral; and EA = Elliptical Area. A/P and M/L RMS are in degrees. A/P and M/L RMS velocity are in degrees per second. Elliptical Area and Path Length are in degrees^2^ and degrees, respectively. An asterisk (*) denotes a significant difference for the non-parametric comparisons between the *No-Task* and *Task* conditions for that metric.

To facilitate comparisons between our previous closed test track study (a driving surrogate characterized as having high physical and moderate functional fidelity) and the current on-road study (naturalistic on-road driving environment), here we report the common balance exercise (Exercise 3) results from our closed test track study (further detailed in Le et al. (2020) [8]). The analysis of normalized balance metrics from the previous closed test track study revealed a significant effect of the *Task* condition on: M/L RMS sway (*p =* 0.023, *Z =* 1.99), M/L RMS sway velocity (*p =* 0.047, *Z* = 1.68), and Path Length (*p =* 0.025, *Z* = 1.96). Figure 2 presents the statistical comparisons, means, and standard errors for all the balance metrics spanning the Urban and closed test track routes (Moderate and Low Acceleration) for Exercise 3. Comparisons between the studies revealed no significant differences in normalized balance metrics across the routes for each *Task* condition. Although the normalized M/L RMS sway velocity for the *Task* condition was greater in the closed test track study, this difference was not significant (*p* = 0.08, *Z =* 1.75). Overall, there were no meaningful differences between the normalized changes in postural sway across the Urban route and the two acceleration levels of the closed test track route.

## 4. Discussion

### 4.1. On-Road Driving Analysis

Across all three balance exercises, postural sway increased following the driving session for each participant, regardless of participant covariates (i.e., age or sex). More specifically, the median values of the normalized metrics were either equivalent to or greater than pre-drive values across the route and *Task* conditions. For the most challenging balance exercise (Exercise 3), all the post-drive balance metrics were observed to increase significantly. Moreover, for Exercises 1 and 2, there were only two balance metrics that did not demonstrate significant increases following a driving session. These balance metrics were the normalized A/P RMS for Exercise 1 and normalized M/L RMS for Exercise 2. However, when comparing across the Urban and Highway routes and *No-Task* and *Task* conditions, there were only minimal differences among the normalized balance metrics. The only significant increase in balance metrics as a function of the *Task* condition was normalized M/L RMS sway for Exercise 3 (37% vs. 22% increase).

For Exercise 3, many balance metrics changed between the two post-drive trials. On average, the relative measures of all balance metrics decreased during the second trial. For the *Task* condition, all changes in the second trial differed significantly from the first trial, with the largest change being a 24% decrease in the median A/P and M/L RMS sway. Moreover, the decreased median value for M/L RMS sway during the second trial (0.83 deg) was similar to the median pre-drive value (0.80 deg), suggesting that post-drive standing balance ability may return to pre-drive levels within a short period of time following egress from a vehicle. However, there were some metrics that increased significantly post-drive but did not significantly decrease in the second post-drive trial. For example, for the *No-Task* condition, we observed significant post-drive increases in A/P and M/L RMS sway and Elliptical Area but no meaningful decreases in those metrics for the second trial, implying that some directional changes in postural sway may be sustained longer than others following a driving session. M/L RMS sway and elliptical area exhibited some of the largest post-drive changes among the six balance metrics (increases of 22% and 35%, respectively, for the *No-Task* condition and increases of 37% and 35% for the *Task* condition) that may explain why changes between the post-drive trials may take longer to recover to pre-drive values. Balance performance that did not fully recover by the second post-drive trial may potentially be a function of the specific postural strategies used for given standing postures [55] and/or explained by potential sensory adaptation [29]. Future work is needed to determine why some metrics were affected more than others and whether in-vehicle exposures lead to measurable sensory adaptations.

Among the published simulated studies that have investigated post-drive balance, Keshavarz et al. (2018) [6] demonstrated that the COP path length of drivers increased during a feet together/eyes open exercise following a simulated driving session on a 6 DOF motion platform. Although a direct comparison was not possible, the directional change of the Path Length (i.e., consistent increase) was similar between this on-road study and the Keshavarz et al. (2018) study, which we report here as a percent change for context. In this on-road study, the percent change between the medians of the non-normalized Path Lengths for Exercise 1 were 6% and 16% for the *No-Task* and *Task* conditions, respectively. In contrast, Keshavarz et al. (2018) [6] found that there was roughly a 21% increase in the COP path length among older adults and a 17% increase among young adults. These similarities between passengers’ and drivers’ postural sway responses warrant additional investigation using direct comparisons.

### 4.2. Comparative Analyses

#### 4.2.1. On-Road vs. Closed Test Track Driving

This on-road study was the first to explore passengers’ standing balance performance following a driving session in a naturalistic on-road environment. The changes in post-drive standing balance performance for Exercise 3 were consistent with the findings from our previous closed test track study [8] that found that all balance metrics increased following an in-vehicle drive. Additionally, there were minimal differences observed between the normalized post-drive postural sway metrics for the closed test track study and this on-road study (only M/L RMS sway velocity was marginally significant at *p =* 0.08), suggesting that the in-vehicle exposures scale similarly. The findings from the pre-post analyses across studies also provide further evidence that the closed test track is a representative experimental platform and surrogate for naturalistic on-road driving exposures.

The closed test track study reported a significant effect of the *Task* condition on more than one balance metric; specifically, normalized M/L RMS sway, normalized M/L RMS sway velocity, and Path Length were greater for the *Task* versus the *No-Task* condition. However, the balance metrics were not observed to differ between the *Task* conditions in this on-road study. Disparities between the findings may be attributed to the differences in the in-vehicle exposures. Although the number of vehicle events and the acceleration associated with each individual vehicle event were standardized, the overall in-vehicle exposure time differed between the closed test track (~20 min) and on-road (~55 min) studies. The association between task performance and increasing post-drive balance metrics observed during the closed test track study may suggest an interaction between task performance and the concentrated driving exposure.

#### 4.2.2. Implications of Post-Drive Standing Balance for Falls

To contextualize the changes in post-drive balance, we chose to compare the RMS of the A/P acceleration with findings from a study by Park et al. (2016) [43] who reported normative RMS trunk acceleration data per decade of age. Following the driving sessions performed with a task, the average RMS A/P acceleration among the younger adult (0.0594 m/s^2^) and older adult (0.0589 m/s^2^) participants reflected measurements likely to be observed in older adults above the age of 60 from the Park et al. (2016) study. In a study conducted by Doheny et al. (2012) [56], RMS A/P acceleration was 20% larger among older adult fallers versus non-fallers. In comparison, the average percent change of post-drive RMS A/P acceleration among older adults in this on-road study was 14% and 42% for the *No-Task* and *Task* conditions, respectively. Hence, following in-vehicle exposures with task performance, the relative change in postural sway suggests that a rider susceptible to balance issues (e.g., an older adult) may be more likely to be at an increased risk for falls [57,58]; an increasingly likely scenario given the anticipated use of AVs and mobility services. However, there is large variability among RMS A/P acceleration data reported in the literature that may be due to a combination of heterogeneity among the sensors, post-processing techniques, and experimental conditions (e.g., vision and stance conditions) used [44,46,48,56], which makes such comparisons challenging.

### 4.3. Limitations

The current study is not without limitations. The balance exercises were performed in different locations. We assessed pre-drive balance outdoors near the laboratory facility, while post-drive balance exercises were performed next to the vehicle immediately upon exiting. However, to partially control for the variation in the visual surroundings, a visual reference target was provided for participants to use during both pre- and post-drive balance exercises. The order of the balance exercises was fixed as well with each subsequent exercise increasing in difficulty; therefore, order may have introduced a learning effect throughout the session. Even with a predetermined route and time of day (midday), we did not fully control for variations in traffic flow given that the driving sessions were affected by real-time traffic conditions. Furthermore, a 60 min driving exposure does not reflect the average time that participants normally spend in a passenger vehicle. Lastly, this analysis only considers standing balance performance before and after a driving session; thus, in-vehicle postural sway of the trunk should be included in future work to close the gap in continuous monitoring and the effects on gait should be explored.

## 5. Conclusions

This on-road study was the first to analyze the relationship between vehicle motion in an on-road setting, task performance, and post-drive balance performance. Postural sway was measured using a personal device-based IMU worn on the lower back. Parameterized using different metrics, postural sway increased post-drive, especially for the most difficult balance exercise. The pre-post changes in normalized postural sway on the Urban route did not differ significantly from a previous study conducted on a closed test track environment. However, the effect of task performance was less significant in this on-road study. Future work should continue to evaluate how an on-road driving exposure affects the standing balance ability of populations already susceptible to falling.

## Figures and Tables

**Table 1:** Normalized values of the pre-post balance metrics for all exercises by *Task* condition. Median values (1st quartile and 3rd quartile) are shown. An asterisk (*) denotes a significant difference between pre-drive and post-drive postural sway for a specific *Task* condition.

	*No-Task*	*Z*	*p*	*Task*	*Z*	*p*
Exercise 1: feet together/eyes open/firm support						
A/P RMS	1.15 (0.72, 1.68)	2.84	* <0.01	1.07 (0.69, 1.78)	2.34	0.019
M/L RMS	1.13 (0.77, 1.48)	2.62	* <0.01	1.23 (0.76, 1.92)	4.04	* <0.001
A/P RMS Velocity	1.03 (0.82, 1.33)	1.87	0.061	1.03 (0.88, 1.34)	2.80	* <0.01
M/L RMS Velocity	1.07 (0.89, 1.34)	3.14	* <0.01	1.10 (0.93, 1.38)	3.97	* <0.001
Elliptical Area	1.13 (0.76, 1.82)	3.29	* <0.01	1.28 (0.75, 2.56)	4.25	* <0.001
Path Length	1.04 (0.90, 1.21)	2.07	0.038	1.03 (0.91, 1.28)	2.68	* <0.01
Exercise 2: feet together/eyes closed/firm support						
A/P RMS	1.07 (0.77, 1.46)	2.14	0.033	1.15 (0.78, 1.57)	3.30	* <0.01
M/L RMS	1.00 (0.71, 1.44)	1.19	0.235	1.08 (0.73, 1.42)	1.88	0.060
A/P RMS velocity	1.03 (0.86, 1.29)	1.95	0.052	1.14 (0.87, 1.30)	3.49	* <0.001
M/L RMS velocity	1.05 (0.88, 1.24)	2.31	0.021	1.15 (0.94, 1.40)	3.57	* <0.001
Elliptical Area	1.13 (0.82, 1.55)	3.21	* <0.01	1.21 (0.74, 1.73)	3.45	* <0.01
Path Length	1.02 (0.90, 1.22)	2.06	0.040	1.06 (0.91, 1.27)	3.13	* <0.01
Exercise 3: feet together/eyes closed/foam support						
A/P RMS	1.14 (0.81, 1.79)	3.16	* <0.01	1.24 (0.79, 2.11)	4.09	* <0.001
M/L RMS	1.22 (0.80, 1.76)	3.70	* <0.001	1.37 (0.95, 1.99)	5.79	* <0.001
A/P RMS velocity	1.23 (1.04, 1.52)	6.15	* <0.001	1.26 (1.01, 1.53)	6.01	* <0.001
M/L RMS velocity	1.38 (1.13, 1.74)	7.01	* <0.001	1.27 (1.05, 1.63)	6.44	* <0.001
Elliptical Area	1.35 (1.08, 2.04)	5.83	* <0.001	1.35 (0.92, 1.98)	5.18	* <0.001
Path Length	1.23 (1.10, 1.55)	7.35	* <0.001	1.28 (1.05, 1.55)	6.99	* <0.001

Abbreviations: RMS = root mean square; A/P = anteroposterior; M/L = mediolateral; and EA = Elliptical Area. A/P and M/L RMS are in degrees. A/P and M/L RMS velocity are in degrees per second. Elliptical Area and Path Length are in degrees^2^ and degrees, respectively.

**Table 2:** Normalized values of the balance metrics for all exercises by *Task* condition. Median values (1st quartile, 3rd quartile) are shown. An asterisk (*) denotes a significant difference between the *No-Task* and *Task* conditions for an exercise.

	*No-Task*	*Task*	*Z*	*p*
Exercise 1: feet together/eyes open/firm support				
A/P RMS	1.15 (0.72, 1.68)	1.07 (0.69, 1.78)	0.90	0.37
M/L RMS	1.13 (0.77, 1.48)	1.23 (0.76, 1.92)	1.80	0.07
A/P RMS velocity	1.03 (0.82, 1.33)	1.03 (0.88, 1.34)	1.03	0.31
M/L RMS velocity	1.07 (0.89, 1.34)	1.10 (0.93, 1.38)	0.65	0.52
Elliptical Area	1.13 (0.76, 1.82)	1.28 (0.75, 2.56)	2.19	0.03
Path Length	1.04 (0.90, 1.21)	1.03 (0.91, 1.28)	1.02	0.31
Exercise 2: feet together/eyes closed/firm support				
A/P RMS	1.07 (0.77, 1.46)	1.15 (0.78, 1.57)	1.14	0.25
M/L RMS	1.00 (0.71, 1.44)	1.08 (0.73, 1.42)	−0.01	0.99
A/P RMS velocity	1.03 (0.86, 1.29)	1.14 (0.87, 1.30)	1.65	0.10
M/L RMS velocity	1.05 (0.88, 1.24)	1.15 (0.94, 1.40)	1.65	0.10
Elliptical Area	1.13 (0.82, 1.55)	1.21 (0.74, 1.73)	0.86	0.39
Path Length	1.02 (0.90, 1.22)	1.06 (0.91, 1.27)	1.54	0.12
Exercise 3: feet together/eyes closed/foam support				
A/P RMS	1.14 (0.81, 1.79)	1.24 (0.79, 2.11)	2.16	0.03
M/L RMS	1.22 (0.80, 1.76)	1.37 (0.95, 1.99)	3.19	<0.01 *
A/P RMS velocity	1.23 (1.04, 1.52)	1.26 (1.01, 1.53)	1.01	0.31
M/L RMS velocity	1.38 (1.13, 1.74)	1.27 (1.05, 1.63)	−0.83	0.41
Elliptical Area	1.35 (1.08, 2.04)	1.35 (0.92, 1.98)	0.86	0.39
Path Length	1.23 (1.10, 1.55)	1.28 (1.05, 1.55)	0.05	0.96

Abbreviations: RMS = root mean square; A/P = anteroposterior; M/L = mediolateral; and EA = Elliptical Area. A/P and M/L RMS are in degrees. A/P and M/L RMS velocity are in degrees per second. Elliptical Area and Path Length are in degrees^2^ and degrees, respectively.

**Table 3:** Changes across post-drive trials described by normalized values for the post-drive trials and grouped by the *Task* condition for Exercise 3 (feet together/eyes closed/foam support). Median values (1st quartile and 3rd quartile) are shown. An asterisk (*) denotes a significant change in the second trial from the first trial.

	*No-Task*			*Task*			*Task* vs. *No-Task*	
	Normalized Trial 2	*Z*	*p*	Normalized Trial 2	*Z*	*p*	*Z*	*p*
A/P RMS	0.95 (0.67, 1.36)	0.48	0.63	0.76 (0.52, 1.16)	−2.35	* 0.02	1.81	0.07
M/L RMS	0.91 (0.58, 1.26)	−0.80	0.42	0.76 (0.52, 1.06)	−4.03	* <0.001	3.35	* <0.001
A/P RMS velocity	0.86 (0.79, 0.98)	−5.46	* <0.001	0.86 (0.72, 0.98)	−5.55	* <0.001	0.98	0.33
M/L RMS velocity	0.83 (0.70, 0.94)	−5.97	* <0.001	0.83 (0.69, 0.96)	−6.07	* <0.001	0.93	0.35
Elliptical Area	0.88 (0.61, 1.13)	−1.86	0.06	0.77 (0.52, 1.05)	−3.81	* <0.001	1.58	0.11
Path Length	0.85 (0.78, 0.95)	−7.03	* <0.001	0.85 (0.76, 0.96)	−6.67	* <0.001	0.63	0.53

Abbreviations: RMS = root mean square; A/P = anteroposterior; M/L = mediolateral; and EA = Elliptical Area. A/P and M/L RMS are in degrees. A/P and M/L RMS velocity are in degrees per second. Elliptical Area and Path Length are in degrees^2^ and degrees, respectively.

## Data Availability

The data presented in this study are available upon reasonable request from the corresponding author. The data are not publicly available due to ongoing analysis.

## Supplementary Materials

The following are available online at https://www.mdpi.com/article/10.3390/s21154997/s1, Figure S1: Map of the scripted Urban route throughout Ann Arbor, Figure S2: Map of the scripted Highway route.
