# Supplementary material for: Post-Drive Standing Balance of Vehicle Passengers Using Wearable Sensors: The Effect of On-Road Driving and Task Performance
Source: Sensors (Basel). 2021 Jul 23;21(15):4997. doi: 10.3390/s21154997 (PMC8347861; doi:10.3390/s21154997)
Supplement: Supplementary file 1 [file sensors-21-04997-s001.zip › sensors-1268842-supplementary.pdf]

Supplementary Materials

Post-Drive Standing Balance of Vehicle Passengers Using Wearable Sensors: The Effect of On-Road Driving and Task Performance

Victor C. Le [1], Monica L. H. Jones [2] and Kathleen H. Sienko [1,*]

[1] Department of Mechanical Engineering, University of Michigan, 2350 Hayward St., Ann Arbor, MI 48109, USA; victle@umich.edu
[2] University of Michigan Transportation Research Institute, University of Michigan, 2901 Baxter Rd., Ann Arbor, MI 48109, USA; mhaumann@umich.edu
* Correspondence: sienko@umich.edu; Tel.: +1-7346478249

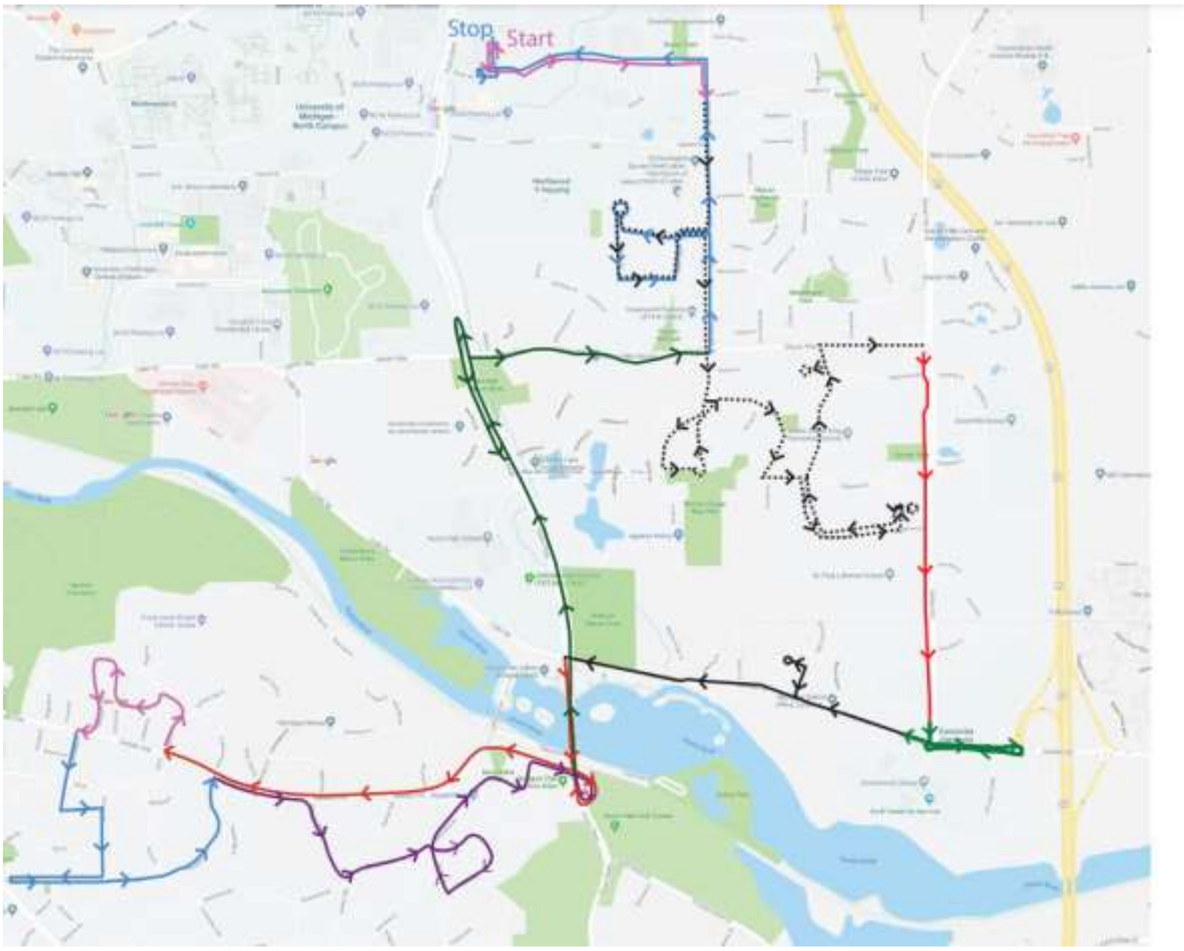

Figure S1. Map of the scripted Urban route throughout Ann Arbor.

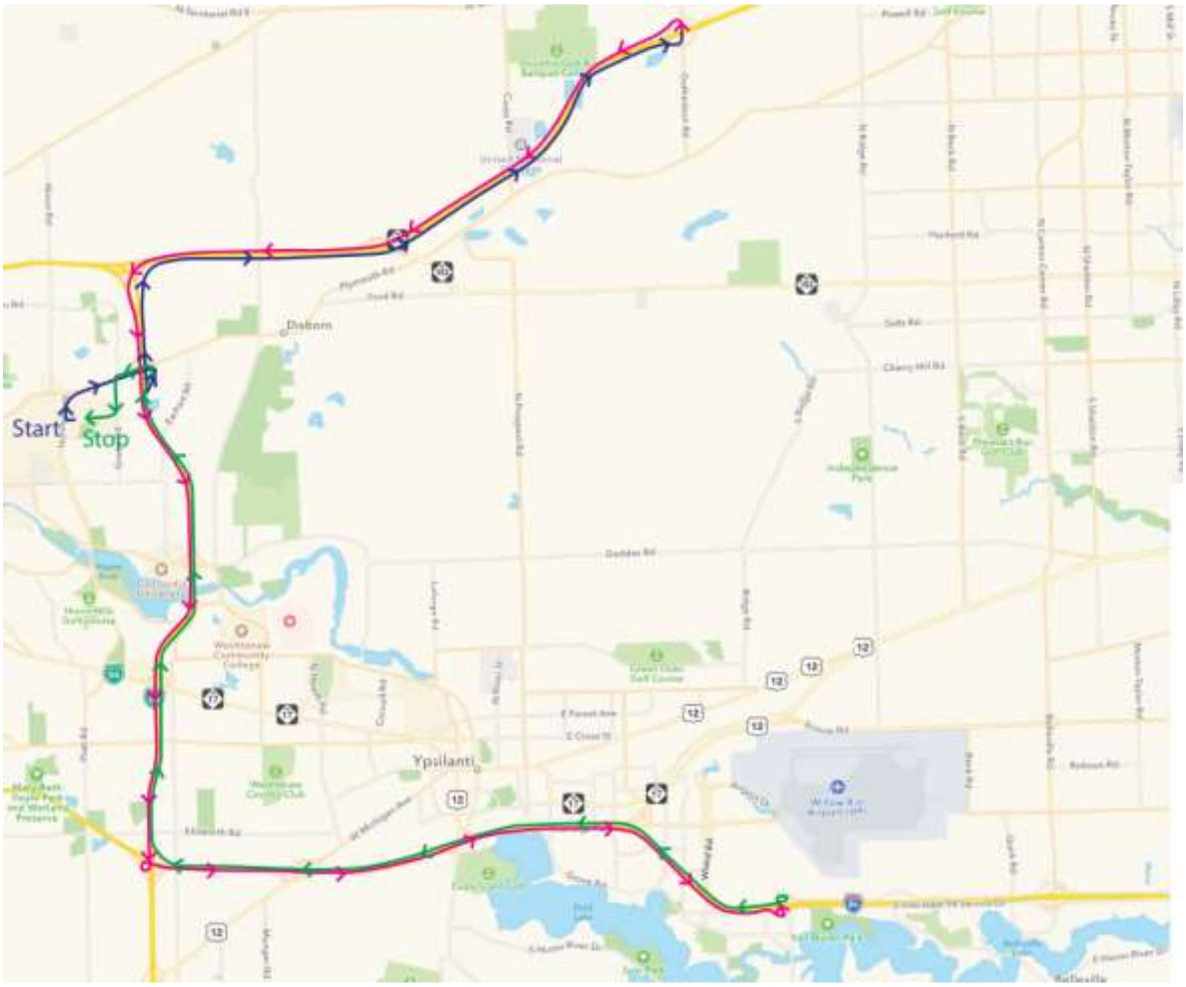

Figure S2. Map of the scripted Highway route.
